# A Systematic Review and Meta-Analysis of Strength Training in Individuals With Multiple Sclerosis Or Parkinson Disease

**DOI:** 10.1097/MD.0000000000000411

**Published:** 2015-01-30

**Authors:** Travis M. Cruickshank, Alvaro R. Reyes, Melanie R. Ziman

**Affiliations:** From the School of Medical Sciences (TMC, ARR, MRZ), Edith Cowan University; and School of Pathology and Laboratory Medicine (MRZ), University of Western Australia, Perth, Australia.

## Abstract

Strength training has, in recent years, been shown to be beneficial for people with Parkinson disease and multiple sclerosis. Consensus regarding its utility for these disorders nevertheless remains contentious among healthcare professionals. Greater clarity is required, especially in regards to the type and magnitude of effects as well as the response differences to strength training between individuals with Parkinson disease or multiple sclerosis.

This study examines the effects, magnitude of those effects, and response differences to strength training between patients with Parkinson disease or multiple sclerosis.

A comprehensive search of electronic databases including Physiotherapy Evidence Database scale, *PubMed*, *EMBASE*, *Cochrane Central Register of Controlled Trials*, and *CINAHL* was conducted from inception to July 2014.

English articles investigating the effect of strength training for individuals with neurodegenerative disorders were selected. Strength training trials that met the inclusion criteria were found for individuals with Parkinson disease or multiple sclerosis.

Individuals with Parkinson disease or multiple sclerosis were included in the study. Strength training interventions included traditional (free weights/machine exercises) and nontraditional programs (eccentric cycling).

Included articles were critically appraised using the Physiotherapy Evidence Database scale.

Of the 507 articles retrieved, only 20 articles met the inclusion criteria. Of these, 14 were randomized and 6 were nonrandomized controlled articles in Parkinson disease or multiple sclerosis. Six randomized and 2 nonrandomized controlled articles originated from 3 trials and were subsequently pooled for systematic analysis. Strength training was found to significantly improve muscle strength in people with Parkinson disease (15%–83.2%) and multiple sclerosis (4.5%–36%). Significant improvements in mobility (11.4%) and disease progression were also reported in people with Parkinson disease after strength training. Furthermore, significant improvements in fatigue (8.2%), functional capacity (21.5%), quality of life (8.3%), power (17.6%), and electromyography activity (24.4%) were found in individuals with multiple sclerosis after strength training.

The limitations of the study were the heterogeneity of interventions and study outcomes in Parkinson disease and multiple sclerosis trials. Strength training is useful for increasing muscle strength in Parkinson disease and to a lesser extent multiple sclerosis.

## INTRODUCTION

Neurodegenerative disorders such as Parkinson disease and multiple sclerosis represent a major medical concern for health professionals and national healthcare bodies.^1^ Both disorders result from progressive neuronal dysfunction and neuronal cell death leading to progressive disability and eventual death.^2^ Classical signs and symptoms customary to both disorders include motor problems, cognitive impairment, behavioral disturbances, and systemic abnormalities.^3–5^

There is no cure and few cost-effective drug agents for treating people with Parkinson disease or multiple sclerosis.^6,7^ Recent advances in understanding the pathogenic mechanisms responsible for each disorder may aid in the identification and development of cost-effective disease-modifying agents in the future.^8^ However, cost-effective treatments, with disease-modifying properties and symptomatic benefits are required in the short term.

Accumulating evidence suggests that strength training is a useful therapy for addressing many of the clinical features that present in individuals with neurodegenerative disorders.^9–11^ By definition, strength training refers to an intervention in which participants train a muscle or group of muscles against an external resistance.^12^ Whereas evidence suggests that lower limb strength training (ie, leg press, knee extension, and knee flexion) is beneficial for individuals with Parkinson disease and multiple sclerosis,^13–19^ consensus regarding the effects, magnitude of those effects, and disease-dependent responses remain contentious. By contrast, the therapeutic utility of strength training is well recognized in the elderly,^20^ individuals with mild cognitive impairment and in those that have suffered a stroke. Health benefits associated with strength training in elderly individuals include improvements in strength,^21,22^ cardiorespiratory capacity,^23^ functional capacity,^23,24^ muscle activity,^25^ body composition,^26^ mood,^27^ cognition,^28,29^ health-related quality of life,^30^ and enhanced hemodynamic activity on functional magnetic resonance imaging tasks.^31^ In individuals who have suffered a stroke, strength training has been found to improve muscular strength, upper and lower limb function and performance on functional tasks.^32–34^ Improvements in selective attention, conflict resolution, associative memory, and regional patterns of functional brain activity have also been observed after strength training in seniors with mild cognitive impairment.^31^

In the last 2 years, 3 systematic reviews have evaluated the effects of strength training in either Parkinson disease or multiple sclerosis.^9,35,36^ Findings from these reviews suggest that strength training is useful for improving muscle strength and some measures of functional capacity in these disorders. Since the publication of these reviews, a number of randomized controlled trials have been published,^9,35,36^ somewhat limiting the informative capacity of previous reviews. Previous systematic reviews have also included trials with confounding supplementary interventions (ie, creatine monohydrate and balance training)^35,36^ as well as trials without a disease control or comparison group.^9,36^ These methodological limitations may have led to an inaccurate appraisal of the effects of strength training as a therapy in individuals with Parkinson disease or multiple sclerosis. It is of vital importance that systematic reviews accurately evaluate experimental therapies like strength training because such documents inform health professionals.

In this systematic review, we provide the most recent evidence to support a robust evaluation of the effect of strength training in people with Parkinson disease or multiple sclerosis. Unlike previous reviews, our study evaluates the effect of strength training alone, in people with Parkinson disease or multiple sclerosis. In addition, our study only selects trials that included individuals with multiple sclerosis or Parkinson disease in the control or comparison group. Moreover, our study evaluates through a meta-analysis, the magnitude of strength improvements in individuals with multiple sclerosis or Parkinson disease in response to strength training. Finally, unlike previous reviews, our study explores whether differences in response to strength training exist between individuals with multiple sclerosis or Parkinson disease.

## MATERIAL AND METHODS

### Search Strategy

A comprehensive search of electronic databases was conducted from inception to July 2014. Electronic searches were performed using Physiotherapy Evidence Database (PEDro) scale, *PubMed*, *EMBASE*, *Cochrane Central Register of Controlled Trials*, and *CINAHL* databases. The search strategy utilized a population, intervention, comparison, and outcome approach.^37^ The population key words were “Parkinson disease,” “multiple sclerosis,” Alzheimer disease, amyotrophic lateral sclerosis, Huntington disease, and spinocerebellar ataxia; the intervention key words were “strength training,” “progressive strength training,” “resistance training,” “weight training,” and “strengthening programs”; and the outcome key words included “strength,” “disease severity,” “gait,” “balance,” “fatigue,” “functional capacity,” “mood,” and “quality of life”. This initial search only found trials on strength training in individuals with Parkinson disease or multiple sclerosis.

As this was a literature review and did not involve the recruitment and assessment of patients, ethical approval was not necessary.

### Eligibility Criteria

Randomized controlled trials and nonrandomized controlled trials that examined the effect of strength training in individuals suffering with multiple sclerosis or Parkinson disease were included in the review. Strength training was defined as an intervention in which participants exercised a muscle or group of muscles against an external resistance.^12^ Eligible studies included those examining the effect of strength training in individuals with multiple sclerosis and Parkinson disease. Exclusion criteria were as follows: case studies; observational studies; studies with healthy controls or healthy comparison groups; and studies employing supplementary intervention therapies in addition to or different from strength training.

### Data Extraction

Two independent authors (T.M.C. and A.R.R) extracted data from the included studies. A specialized extraction form was designed and recorded the following methodological details for each study as described below.

Publication details: authors and year of publication; details of the study: study design and number of participants, experimental and control interventions, and reported outcomes (controls and experimental); participant characteristics: disease population, disease status, and age; specific intervention details: intervention groups, mode of strength training, targeted anatomical regions, setting in which the study was conducted, level of supervision, duration of the intervention (weeks), frequency of strength training, specific exercises employed, exercise intensity, number of sets and repetitions performed for each exercise, rest taken between sets and exercises, and the progression method used for strength training interventions; moderator variables: participant retention and dropouts, participant adherence, and adverse effects associated with strength training.

Corresponding authors of studies were contacted as necessary for supplementary information not detailed in the publication. In cases wherein authors did not respond or did not provide supplementary methodological information pertaining to their publication, a not reported statement was assigned.

### Quality Assessment

All articles that satisfied the predefined inclusion criteria were independently rated for quality by 2 reviewers (T.C. and A.R.) using the PEDro scale.^38^ The PEDro scale is an 11 points scale designed to examine the methodological quality of intervention studies. The scale evaluates the following methodological aspects: specific eligibility criteria, randomization allocation, concealed allocation, baseline demographic similarities, participant blinding, therapist blinding, outcome assessor blinding, whether more than 85% of participants completed follow-up for at least 1 primary outcome, intention to treat analysis, between group statistical comparisons, and point estimates and variability for at least one of the primary outcome measures. When rating each study, only criteria 2 and 11 are considered for the PEDro scale. Initial discrepancies between the independent authors were resolved by consensus. In instances wherein discrepancies could not be resolved, a final decision was made by another independent author (M.Z.).

### Data Analysis and Synthesis

For analysis, studies were categorized according to disease. The heterogeneity of populations and extensive variety of reported outcomes prevented a meta-analysis for all outcomes, with the exception of strength. Whereas 15 articles reported on strength as an outcome,^13–16,18,19,39–45^ 3 articles by Dalgas et al^16,18,19^ and 2 articles by Dibble et al^42,43^ appeared to originate from the same trial. Strength data from 3 articles by Dalgas et al^16,18,19^ were pooled together into a single effect size for a better interpretation of the effects of strength training on strength as an outcome. Standardized effect sizes were calculated for the meta-analysis using pre- and poststrength mean values for each group (intervention and comparison) (Hedges and Olkin, 1985). Effect sizes were corrected for the magnitude of sample size of each study as suggested by Hedges and Olkin (1985). The risk of publication bias in trials was examined statistically using the egger regression test, with a significant publication bias considered to be *P* ≤ 0.10. All statistical analyses were performed using STATA 9.1 (StataCorp LC, Texas, USA).

## RESULTS

### Articles Included

**FIGURE 1:**
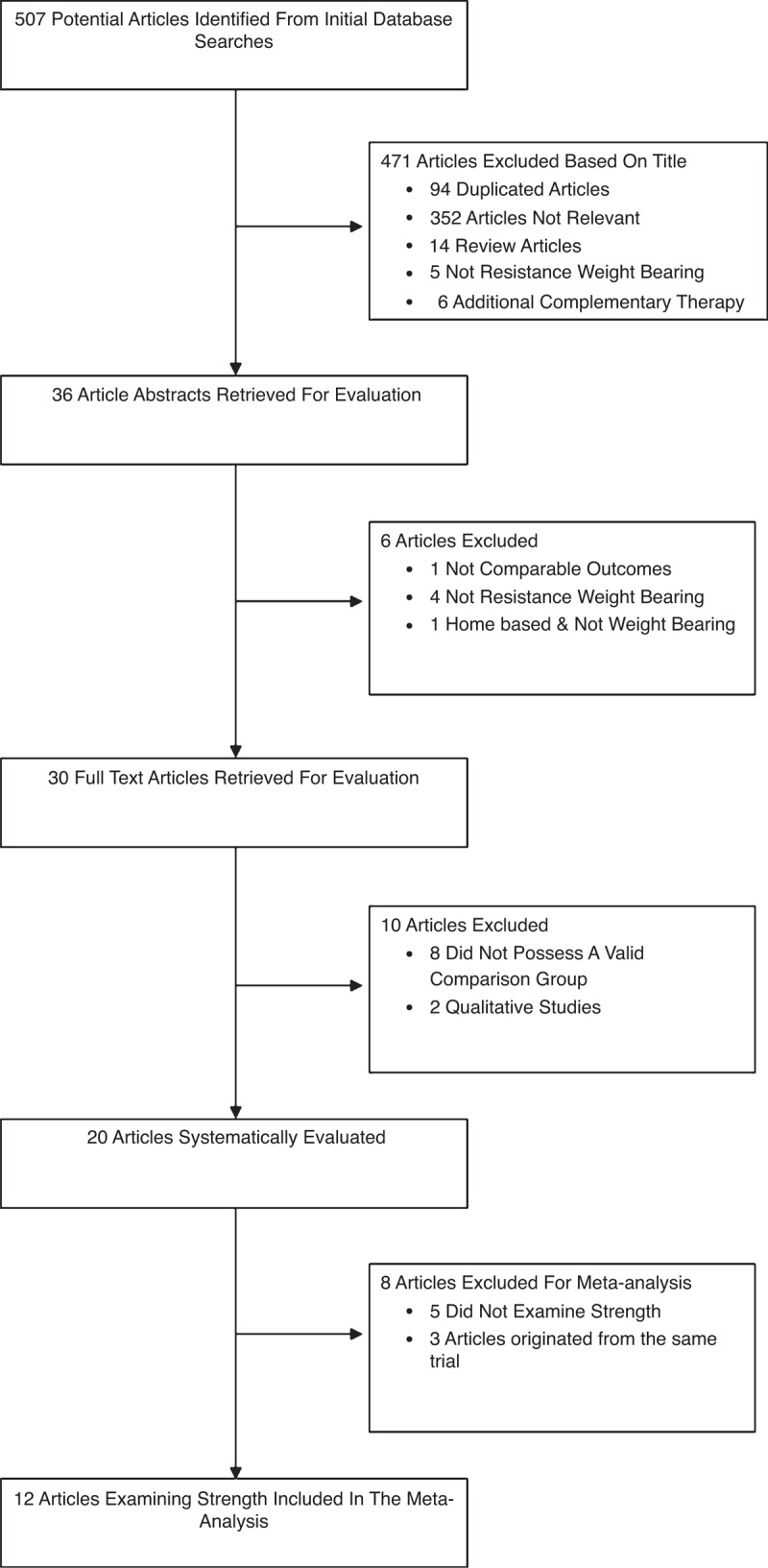
Flowchart for selection of trials included in the systematic review and meta-analysis.

The database search strategy and results are presented in Figure 1. Five hundred seven articles were identified by the initial search strategy. Four hundred seventy one of the identified articles were excluded based on their title. The abstracts of the remaining 36 articles were evaluated and 6 articles were excluded (Figure 1). Full texts of the remaining 30 articles were retrieved and reviewed, resulting in the exclusion of 10 articles (Figure 1). Of the 20 articles included in the systematic review, 8 appeared to originate from 3 separate trials. Subsequently, the extracted and reviewed data is representative of 15 independent trials.

### Methodological Quality

The methodological quality of included trials varied considerably in both Parkinson disease and multiple sclerosis populations. PEDro scores ranged from 4 to 8 points in both Parkinson disease^13,14,40,42–44,46–49^ and multiple sclerosis trials^15–19,39,41,45,50,51^ (Table 1).

**TABLE 1:**
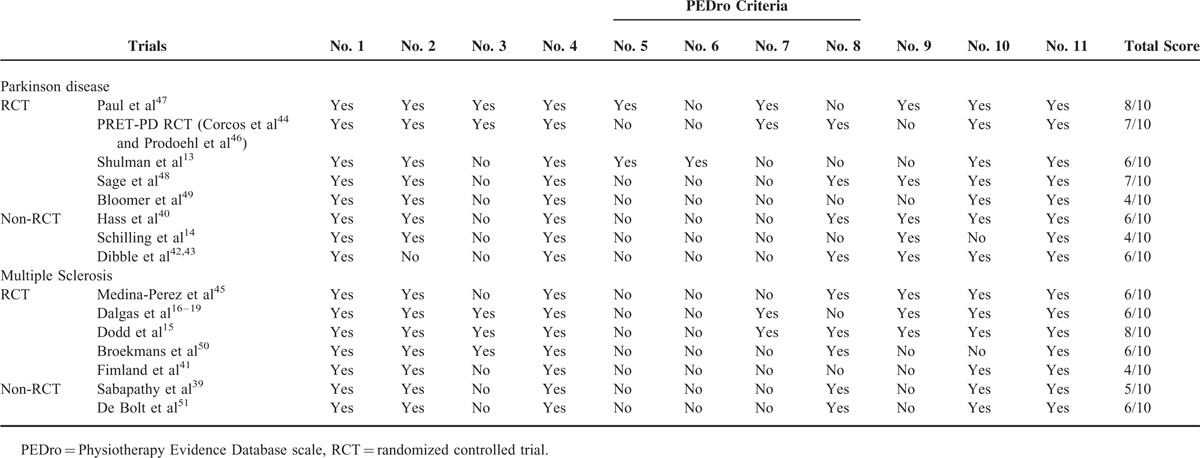
Trial Inclusions Rated According to the Physiotherapy Evidence Database Scale

### Participants Characteristics

The number of trials included was 8 in Parkinson disease^13,14,40,42–44,46–49^ and 7 in multiple sclerosis.^15–19,39,41,45,50,51^ Disease population, study design, number of participants, stage of disease, mean age and standard deviation, trial intervention, and trial outcomes are shown in Tables 2 and 3 .

**TABLE 2:**
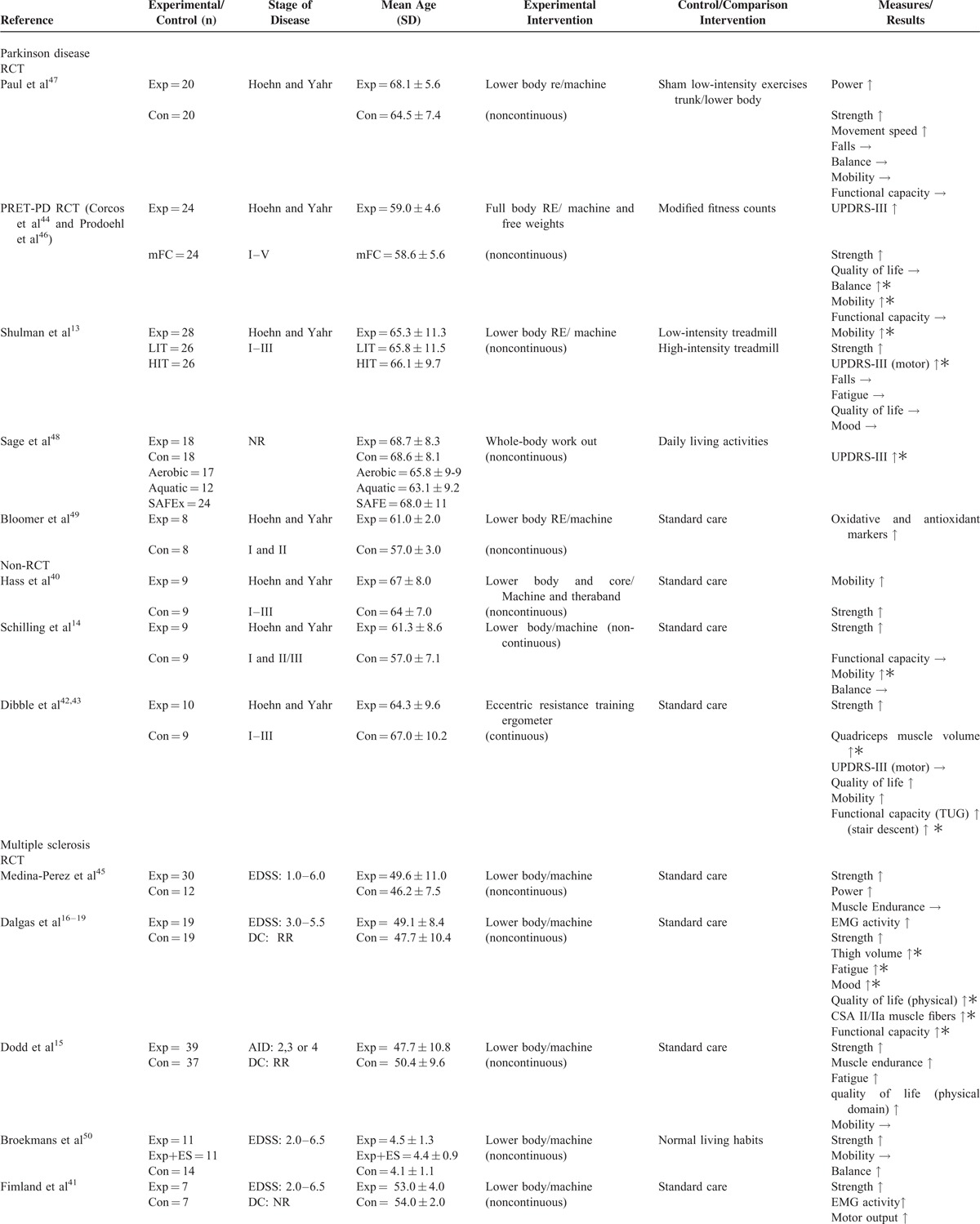
Overview of Trials of Strength Training Interventions in Individuals With Parkinson Disease or Multiple Sclerosis

**TABLE 2 (Continued):**
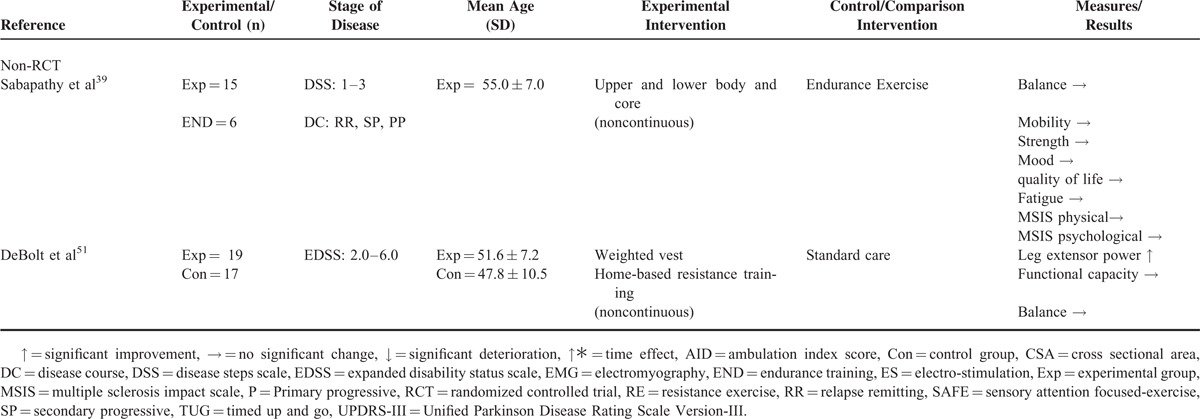
Overview of Trials of Strength Training Interventions in Individuals With Parkinson Disease or Multiple Sclerosis

**TABLE 3:**
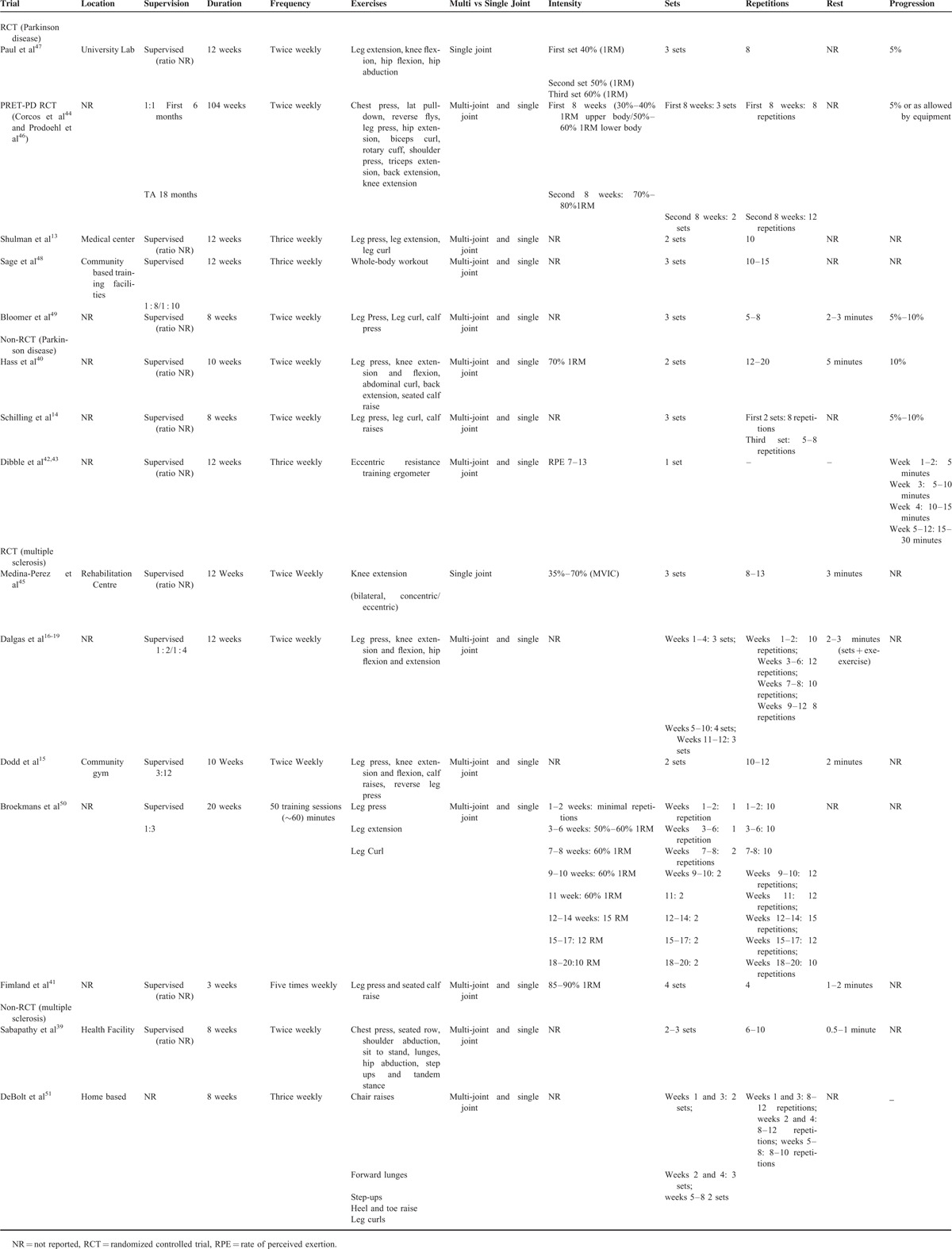
Summary Details of the Specific Strength Training Interventions Used in Parkinson Disease or Multiple Sclerosis Trials

### Intervention Characteristics 

Of the 8 trials conducted in individuals with Parkinson disease^13,14,40,42–44,46–49^ (5 randomized controlled trials^13,44,46–49^ and 3 nonrandomized controlled trials^14,40,42,43^), 5 used lower body strength training interventions,^13,14,42,43,47,49^ 2 used a full-body strength training intervention,^44,46,48^ and 1 used a lower body and core strength training intervention^40^ (Tables 2 and 3 ). Training protocols ranged from 2 to 24 months of twice to thrice weekly training.^13,14,40,42–44,46–49^ Only 2 trials conducted in individuals with Parkinson disease reported on the level of supervision for strength training interventions.^44,46,48^

Of the 7 trials conducted in multiple sclerosis^15–19,39,41,45,50,51^ (5 randomized controlled trials^15–19,41,45,50^ and 2 nonrandomized controlled trials^39,51^), 5 trials trained the lower body^15–19,41,45,50^ and 2 trials trained the full body^39,51^ (Tables 2 and 3 ). Intervention protocols utilized in multiple sclerosis trials ranged from 3 weeks to 6 months of 2 to 5 times weekly training.^15–19,39,41,45,50,51^ Of the 7 trials conducted in individuals with multiple sclerosis, only 3 trials reported on the level of supervision for strength training interventions.^15–19,50^

### Risk of Bias

Statistical examination using the egger regression test revealed no publication bias (*P* = 0.131).

### Intensity and Progression of Strength Training

Two randomized^44,46,47^ and 2 nonrandomized controlled trials^40,42,43^ conducted in Parkinson disease reported on the intensity of strength training performed throughout the intervention, whereas 3 randomized controlled trials^41,45,50^ reported on the intensity of strength training in multiple sclerosis. The progression of strength training was reported by 3 randomized^44,46,47,49^ and 3 nonrandomized controlled trials^14,40,42,43^ in Parkinson disease. In contrast, there were no trials that reported on the progression of strength training in multiple sclerosis.

### Participant Retention, Adherence, and Adverse Events

Participant retention ranged from 75% to 100% in Parkinson disease trials^13,14,40,42–44,46–49^ and from 73.3% to 100% in multiple sclerosis trials^15–19,39,41,45,50,51^ (Table 4). Four trials in multiple sclerosis ([Medina-Perez et al^45^ strength training group 95.4%; control group not reported], [Dodd et al^15^ strength training group 92%; control group 62%], [Broekmans et al^50^ ∼99% all groups] and [DeBolt et al^51^ strength training group 95%]), and 1 trial in Parkinson disease reported on participant adherence (Paul et al^47^ strength training group 84.1%; control group 94.1%) (Table 4). Five trials in Parkinson disease^13,40,44,46–48^ and 6 trials in multiple sclerosis^15–19,39,41,45,50^ reported on adverse events,^13,40,44,46–48^ with only minor or clinically unrelated medical issues reported (Table 4).

**TABLE 4:**
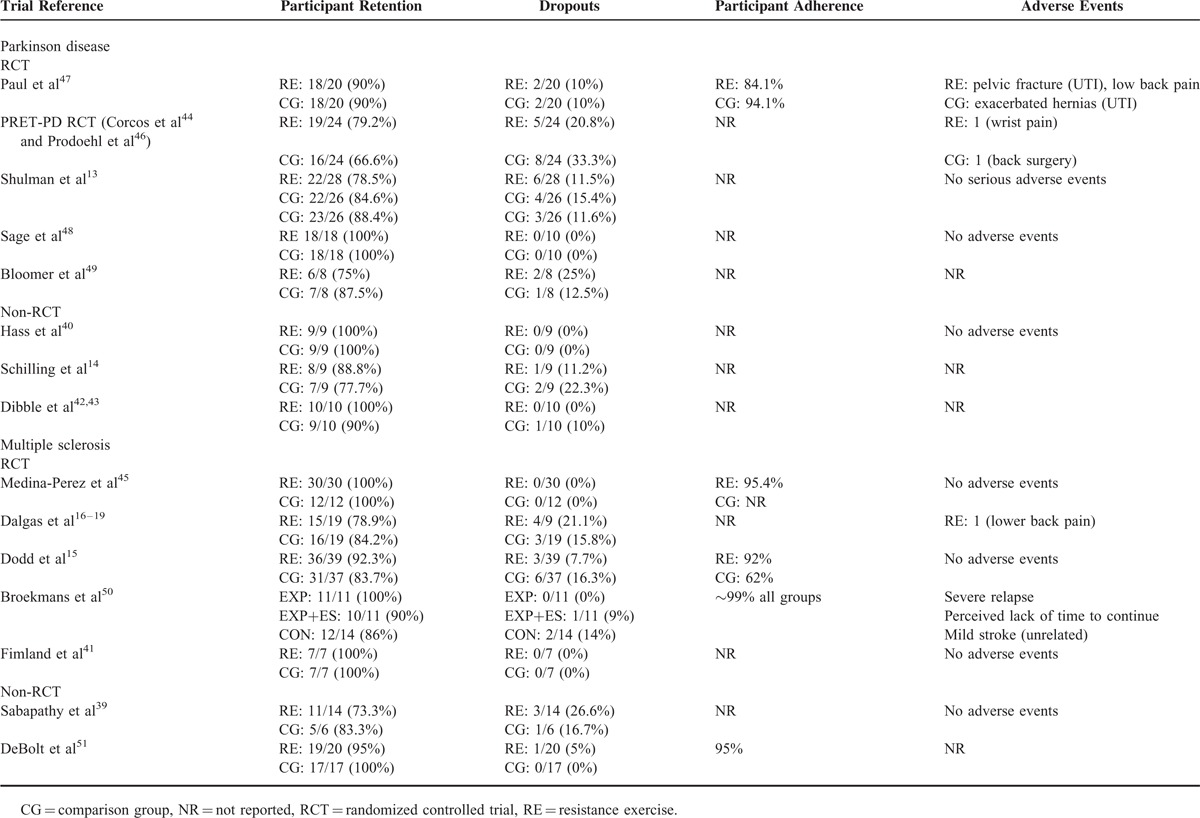
Summary of Retention, Adherence and Adverse Events in Parkinson Disease or Multiple Sclerosis Strength Training Trials

### Outcomes Measures

#### Strength As an Outcome Measure in Parkinson Disease

Three randomized controlled trials evaluated the effect of strength training on strength in people with Parkinson disease.^13,44,47^ Strength was evaluated across trials using 1 repetition maximum (1RM) and maximum voluntary isometric contraction (MVIC) protocols with torque transducers, pneumatic resistance machines, and dynamometers. Corcos et al^44^ found a significant improvement in elbow flexor muscle strength (1RM, 15%) in the strength training group, while off medication, after 24 months of upper and lower body resistance training. No significant differences in strength were found for the control group in this trial. Shulman et al^13^ in another trial found a significant improvement in leg press and leg extension strength (1RM, 16%) in individuals within the strength training group, but not in the high or low intensity treadmill training groups, after 3 months of thrice weekly resistance training. Paul et al^47^ also reported a significant improvement in lower limb strength (1RM, leg extension, 14.6%; knee flexion, 18.6%; hip flexion, 39.8%; hip abduction, 33.9%) and power (leg extension, 17.3%; knee flexion, 20.6%; hip flexion, 46.3%; hip abduction, 43.1%) in the strength training group in comparison to the sham comparison group after 12 weeks of lower body resistance training.

Three nonrandomized controlled trials also evaluated the effect of strength training on strength and found significant improvements.^14,40,42,43^ Hass et al,^40^ after 10 weeks of twice weekly lower body strength training, found a significant improvement in knee extension (1RM, 76%) and knee flexion (1RM, 57%) strength in the intervention group, but not in the control group. Schilling et al^14^ in another trial reported a significant improvement in leg press strength (1RM, 22%) in the intervention group, whereas the control group showed no significant differences. Dibble et al^42,43^ similarly reported a significant improvement in quadriceps muscle strength (MVIC) in the more (average torque 23%; peak torque 18%) and less-affected leg (average torque 16%; peak torque 83.2%) in the strength training intervention group only.

#### Strength As an Outcome in Multiple Sclerosis

Five randomized controlled trials reported on strength as an outcome after strength training,^15,16,18,19,41,45,50^ with all 5 trials reporting significant improvements in strength. Strength was evaluated across trials using MVIC, maximum voluntary dynamic contraction, and 1RM strength protocols with pneumatic resistance machines, dynamometers and the Leg Extensor Power Rig. Medina-Perez et al^45^ reported a significant improvement in knee extension strength (MVIC, 7.7%) and power (40% MVIC, 15.6%) in the intervention group, but not in the control group after 12 weeks of strength training. Significant improvements in leg press strength (1RM, 15%) in the intervention group, but not the control group were also reported by Dodd et al^15^ after strength training. Broekmans et al^50^ in line with Medina-Perez et al,^45^ reported significant improvements in isometric strength in the knee flexors and extensors (MVIC, average knee extension 45° change: 10.8, average knee extension 90° change: 10, average knee flexor 45° change: 4, average knee flexion 90° change: 2.3) in the intervention group as a result of strength training. In another trial, Dalgas et al^16,18,19^ reported significant improvements in isokinetic, isometric, and angular impulse knee extensor and flexor strength in the intervention group ([Dalgas et al,^19^ MVIC at 70° knee flexion; knee extension: 13.2%, knee flexion: 13.8%], [Dalgas et al^18^; maximum voluntary dynamic contraction, knee extension 90°: 4.5%; knee extension 180°:10.2%; knee flexion 90°: 21.3%; knee flexion 180°: 18.6%], [Dalgas et al,^16^ MVIC, knee extension: 15.7%, knee flexion: 21.3%]), but not in the control group as a result of resistance training. Dalgas et al^16^ additionally reported a significant improvement in leg press strength. Fimland et al^41^ in another trial reported a significant improvement in plantar flexion strength (MVIC, 36%) in the strength training intervention group, but not in the control group. In a nonrandomized controlled trial, DeBolt et al^51^ reported a significant improvement in leg extensor power (24%) in the intervention group, whereas the disease control group showed no changes after strength training.

In addition to muscle strength, significant study-specific improvements in gait, clinical disease progression, functional capacity, quality of life, oxidative biomarkers, mood, fatigue, falls, skeletal muscle volume, and electromyography activity were observed after strength training in individuals with multiple sclerosis or Parkinson disease.^13–19,39–51^

### Parkinson Disease Measures

#### Unified Parkinson Disease Rating Scale Version 3

Three randomized^13,44,48^ and 1 nonrandomized controlled trial^42^ conducted in Parkinson disease evaluated the effect of strength training on clinical disease progression using the Unified Parkinson Disease Rating Scale Version 3. Corcos et al^44^ reported a significant improvement on the Unified Parkinson Disease Rating Scale Version 3 in the intervention group (7.4 point decrease), but not in the control group after 24 months of strength training. Shulman et al^13^ in another study similarly reported a significant improvement on the motor subscale of the Unified Parkinson Disease Rating Scale Version 3 in the strength training group. Furthermore, Sage et al^48^ found a significant improvement on the Unified Parkinson Disease Rating Scale Version 3 in the strength training group. Dibble et al^42^ by contrast found no improvement on the Unified Parkinson Disease Rating Scale Version 3 in the intervention group after strength training.

#### Functional Mobility

Three randomized^13,46,47^ and 3 nonrandomized controlled trials^14,40,42,43^ evaluated the effect of strength training on mobility in individuals with Parkinson disease. Mobility was assessed across trials using the 10 meter timed walk test, 6 minute walk test, 50 feet walk test and timed up and go. Paul et al^47^ did not report significant changes in mobility after strength training. In contrast, Prodoehl et al^46^ and Shulman et al^13^ found significant improvements in mobility as a result of strength training. The 3 nonrandomized controlled trials^12,39,41,42^ that reported on mobility as an outcome also documented improvements.

#### Balance

Two randomized^46,47^ and 2 nonrandomized controlled trials^14,39^ examined the effect of strength training on balance outcomes in Parkinson disease. Balance was evaluated across trials using a variety of outcomes including the single leg stance, choice stepping task, berg balance scale, functional reach test, 5 time sit to stand test, and the activities-specific balance confidence scale. Paul et al^47^ did not find a significant improvement in balance as a result of strength training. Prodoehl et al^46^ by contrast reported a significant improvement in balance after strength training. Both nonrandomized controlled trials^14,39^ were unable to find a significant improvement in balance after strength training.

#### Functional Capacity

One randomized trial^44^ examined the effect of strength training on functional capacity. Corcos et al^44^ assessed functional capacity using the modified Physical Performance Test and reported no significant changes after strength training in the intervention or control group.

#### Quality of Life

Two randomized^13,44^ and 1 nonrandomized controlled trial^42^ evaluated the effect of strength training on quality of life. All 3 trials assessed quality of life using the 39-Item Parkinson Disease Questionnaire. Both randomized controlled trials^10,11^ did not report a significant improvement in quality of life after strength training. Dibble et al^42^ by contrast reported a significant improvement in quality of life in the intervention group after strength training.

#### Oxidative and Antioxidant Markers

One randomized controlled trial^49^ in Parkinson disease measured changes in blood oxidant and antioxidant marker levels and reported significant increases in antioxidant marker levels (superoxide dismutase [9.9%] and glutathione peroxidase [1.8%]) and a significant reduction in oxidative stress marker levels (malondialdehyde [15%] and hydrogen peroxide [16%]).

#### Mood

One randomized controlled trial^13^ evaluated the effect of strength training on mood in Parkinson disease. Shulman et al^13^ found no significant changes in mood after strength training using the Beck Depression Inventory.

#### Fatigue

One randomized controlled trial^13^ evaluated the effect of strength training on fatigue in Parkinson disease. Shulman et al^13^ used the 16-item Parkinson Fatigue Scale and found no significant change in fatigue after strength training in the strength training intervention group or high- and low-intensity treadmill intervention groups.

#### Falls

Two randomized controlled trials^11,45^ evaluated the effect of strength training on falls in people with Parkinson disease.^13,47^ Falls were assessed using the New Freezing of Gait Questionnaire^47^ and Falls Efficacy Scale.^13^ No trial reported a significant effect on falls outcomes after strength training.

#### Skeletal Muscle Volume

One nonrandomized controlled trial^43^ evaluated the effect of strength training on quadriceps muscle volume in Parkinson disease. Dibble et al^43^ found a significant increase in quadriceps muscle volume using magnetic resonance imaging after strength training in the intervention group only.

### Multiple Sclerosis

#### Functional Mobility

Two randomized^15,50^ and 2 nonrandomized controlled trials^39,51^ evaluated the effect of strength training on functional mobility in multiple sclerosis. Functional mobility was assessed across trials using the 2 minute walk test, 10 meter walk test, timed 25 foot walk and timed up and go. No trial reported a significant improvement in mobility as a result of strength training.

#### Balance

One randomized^50^ and 2 nonrandomized^39,51^ controlled trials evaluated the effect of strength training on balance in multiple sclerosis. Balance was evaluated across trials using the Functional Reach Test,^39,50^ Four Square Step Test,^39^ and Accusway^PLUS^ force platform.^51^ Broekmans et al^50^ reported a significant improvement in balance in the intervention group only as a result of strength training. However, Sabapathy et al^39^ and DeBolt et al^51^ did not find significant improvements in balance after strength training.

#### Functional Capacity

One randomized controlled trial^16^ evaluated the effect of strength training on functional capacity outcomes in multiple sclerosis. Dalgas et al^16^ reported a significant improvement in functional capacity (computed as 1/4 [chair stand test _post_/chair stand test _pre_] + [stair climb test _post_/stair climb test _pre_] + [10 meter walk test _post_ /10 meter walk test _pre_] + [6 minute walk test _post_ / 6 minute walk test _pre_] × 100) as a result of strength training.

#### Quality of Life

Two randomized^15,17^ and 1 nonrandomized controlled trial^39^ reported on quality of life outcomes after strength training in multiple sclerosis. Quality of life was assessed across trials using the Short Form-36^17,39^ and the World Health Organisation Quality of Life-BREF.^15^ Dodd et al^15^ and Dalgas et al^17^ reported a significant improvement in quality of life in the intervention group as a result of strength training. In contrast, Sabapathy et al^39^ found no significant improvement in quality of life after strength training.

#### Electromyography Activity

Two randomized controlled trials^17,40^ assessed the effect of strength training on electromyography activity during maximum voluntary isometric contractions. Dalgas et al recorded surface electromyography signals from the Vastus Lateralis, Rectus Femoris, and Semitendinosus during maximal voluntary isometric contractions of the knee flexors and extensors (assessed at 70° knee flexion), using bipolar electrodes. The upper electrode of each pair was placed at the midpoint between the Spina Iliaca anterior superior and patellar basis. After 12 weeks of strength training, Dalgas et al found significant improvements in maximal isometric (μV) knee extension and knee flexion activity (semitendinosus: 27.6%; vastus lateralis: 27%; rectus femoris: 28%) in the intervention group, but not the control group. Fimland et al^41^ recorded surface electromyography activity during maximum voluntary isometric contractions of the plantar flexors (ankle positioned at 90°), using bipolar surface electrodes placed according to Surface Electromyography for the Noninvasive Assessment of Muscles recommendations. Fimland et al^41^ reported significant improvements (15%) in surface electromyography activity of the plantar flexors after 3 weeks of strength training in the intervention group in comparison to the control group.

#### Skeletal Muscle Volume and Architecture

Only 1 randomized controlled trial^18^ measured changes to thigh volume, muscle fiber numbers, type, and size. Muscle biopsies of the vastus lateralis (middle portion) were taken to assess changes in muscle fiber number, type, and size. Dalgas et al^18^ reported a significant increase in the cross sectional area of type II and IIa vastus lateralis muscle fibers after strength training in the intervention group only.

#### Fatigue

Two randomized^15,17^ and 1 nonrandomized controlled trial^39^ evaluated the effect of strength training on fatigue in multiple sclerosis. Fatigue was assessed across trials using a variety of outcomes including the Modified Fatigue Scale and Fatigue Severity Scale, Multidimensional Fatigue Inventory. Dodd et al^15^ reported a significant improvement in the level of fatigue experienced (24%) after 10 weeks of twice weekly strength training. Similar findings were reported by Dalgas et al,^17^ who reported a 10% improvement in the level of fatigue experienced after strength training. Sabapathy et al^39^ also reported a significant improvement in the level of fatigue experienced as a result of strength training.

#### Mood

One randomized^17^ and 1 nonrandomized controlled trial^39^ examined the effect of strength training on mood outcomes in multiple sclerosis. Dalgas et al^17^ reported significant improvements (−2.4 points) in mood using the Major Depression Inventory as a result of strength training. In contrast, Sabapathy et al^39^ found no significant changes in mood using the Beck Depression Inventory after strength training.

#### Muscle Endurance

Two randomized controlled trials^15,45^ evaluated the effect of strength training on muscle endurance in multiple sclerosis. Medina-Perez et al^45^ measured muscle endurance as the maximum number of repetitions that a participant could perform during a single set of knee extension using a load of 40% of the maximum voluntary isometric contraction, whereas Dodd et al^15^ measured endurance by counting the number of repetitions that a participant could complete on the seated leg press and reverse leg press using a load of 50% of 1RM. Medina-Perez et al^45^ did not find a significant change in muscle endurance in the intervention or control group after strength training. In contrast, Dodd et al^15^ reported a significant improvement in muscle endurance in the intervention group relative to the control group after strength training.

## DISCUSSION

This review found that strength training is useful for improving muscle strength in Parkinson disease and to a lesser extent multiple sclerosis. Evidence also showed that strength training is helpful for improving clinical measures of disease progression and mobility in Parkinson disease. However, the evidence is unclear regarding the efficacy of strength training on falls, quality of life, fatigue, functional capacity, and balance in Parkinson disease. In multiple sclerosis, strength training was also found to improve fatigue, quality of life, muscle power, electromyography activity, and functional capacity. However, its effect on balance and mood remains equivocal.

**FIGURE 2:**
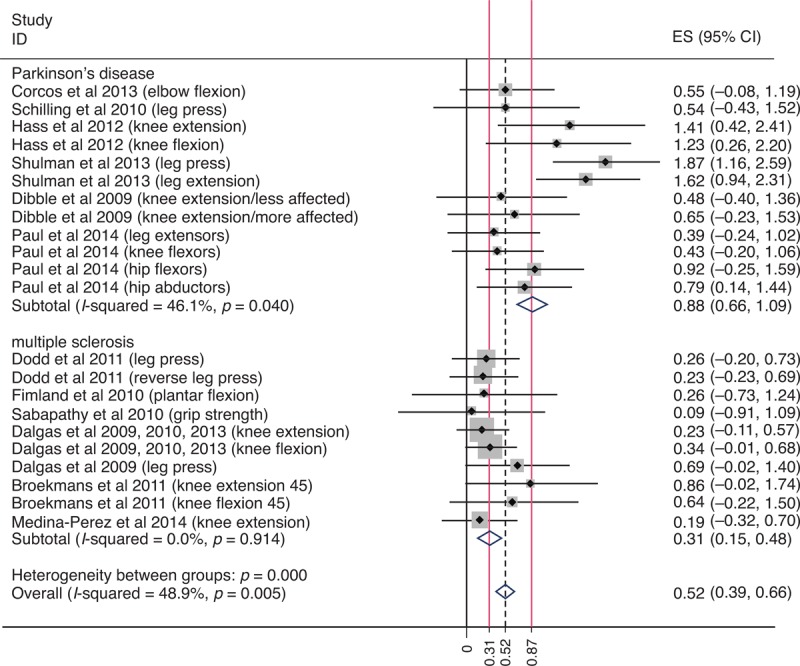
Meta-analysis of trials that measured muscle strength.

An increase in strength was the most consistently reported benefit of strength training in people with Parkinson disease and multiple sclerosis. A meta-analysis of the extracted strength data revealed that strength training had a larger effect on strength in people with Parkinson disease (d = 0.87) than multiple sclerosis (d = 0.33) (Figure 2). Different pathological mechanisms underpinning impairments in strength in each disease are likely to account for this discrepancy. For instance, impairments in strength in multiple sclerosis are thought to be mediated by central^52,53^ (spinal and supraspinal mechanisms) and muscular deficits,^54–56^ whereas in Parkinson disease impairments in strength are thought to result from central deficits only.^57–59^ This finding suggests that strength training may only produce meaningful benefits in strength in people with Parkinson disease.

Strength training trials in Parkinson disease also reported improvements in mobility. The improvements were reported on short and longer duration mobility assessments, suggesting that strength training has a favorable effect on multiple aspects of mobility. This finding is consistent with the supposition that muscle strength strongly predicts mobility in people with Parkinson disease.^60,61^ Surprisingly, no improvements in mobility were reported in individuals with multiple sclerosis after strength training. This finding was unexpected, as the strength training interventions in Parkinson disease and multiple sclerosis trials, for the most part, used similar training frequencies (2–3 times per week), resistance exercises (leg press, knee extension, knee flexion, and calf raises), and sets per exercise (2–3). This may indicate that strength training is not capable of improving mobility in individuals with multiple sclerosis. The inability to improve mobility may be explained by the smaller improvements in strength observed in individuals with multiple sclerosis. Indeed, recent findings show that muscle strength significantly predicts performance on mobility tasks in individuals with multiple sclerosis.^62^ Alternatively, it is possible that the strength training interventions used in the multiple sclerosis trials were unable to provide a stimulus sufficient to improve mobility in multiple sclerosis, and perhaps more intense or specific training interventions may be required.

In addition, strength training was found to have a positive effect on disease progression in people with Parkinson disease (Unified Parkinson Disease Rating Scale-Version 3). Interestingly, improvements in disease progression were observed in a cohort with mild-to-advanced disability that were not on medication, suggesting that strength training alone may be capable of positively impacting on disease progression in individuals at all stages of Parkinson disease. The positive effect of strength training on disease progression may have been mediated by favorable central changes. For instance, recent evidence shows that repetitive force generation increases neuronal activation in the basal ganglia, thalamus, parietal cortex, cerebellum, and motor cortex.^63–66^ Furthermore, emerging evidence has shown that exercise interventions can increase regional brain volume and structural connectivity in patients with Parkinson disease and other neurodegenerative disorders.^67–70^ Further studies are required to confirm the latter remarks.

In multiple sclerosis trials, improvements in strength were accompanied by significant improvements in fatigue, quality of life, muscle power, maximal electromyography activity, and functional capacity. The reported improvements in fatigue are of clinical interest given that 33%–75% of individuals with multiple sclerosis suffer from fatigue.^71–73^ Nevertheless, this finding was not surprising, given that exercise has previously been reported to improve fatigue in multiple sclerosis.^74^ The improvements in fatigue may in part explain the benefits observed in quality of life, especially considering that fatigue is an important predictor of quality of life in people with multiple sclerosis.^75,76^ The increases in muscle power and maximal electromyography activity are consistent with the observed improvements in strength. The reported improvements in lower limb strength, fatigue, and muscle power likely contributed to the improvement in functional capacity documented by Dalgas et al.^16^ Indeed, recent findings have shown that strength,^77^ fatigue,^78^ and muscle power^61^ significantly influences functional capacity in individuals with multiple sclerosis and other neurodegenerative disorders.

It is important to note that most trials included in this systematic review recruited individuals with mild-to-moderate disability. The higher level of disability in individuals at advanced stages of Parkinson disease or multiple sclerosis may have led researchers to only include individuals at early-to-middle stages of both diseases. The same level of benefits after strength training may not be possible in individuals at more advanced stages of Parkinson disease or multiple sclerosis. Future trials assessing the effect of strength training in individuals with Parkinson disease and multiple sclerosis with a severe level of disability are therefore warranted.

In general, the trials displayed adequate methodological quality, with PEDro scores ranging from 4 to 8 in both diseases. The major methodological shortcomings found using the PEDro scale included a failure to report concealed allocation (criteria 3), participant blinding (criteria 5), therapist blinding (criteria 6), and outcome assessor blinding (criteria 7). It is important to acknowledge that it is often not possible to blind participants or therapists to exercise or group allocation.^79^ Trial scores generated using the PEDro scale may therefore underestimate the quality of evidence.

In addition to evaluating trials using the PEDro scale, we also performed a critical appraisal of specific intervention characteristics important to strength training trials. This appraisal found that specific intervention characteristics were typically well detailed, with the exception of the level of supervision and strength training intensity. The lack of data reported on the level of supervision and the intensity of strength training performed is of concern in particular, as a high level of supervision as well as an appropriate intensity of strength training is required to maximize therapeutic benefits and avoid potential injury.^80^ The poor level of reporting on strength training progression in multiple sclerosis trials is also concerning, given that modulating the progression of strength training is important to avoid injury and training plateaus.^81^ The inadequate reporting of participant adherence in both disease populations was also worrisome, as it does not enable internal and external examination of what dose of strength training is required to maximize therapeutic benefits and avoid injury in such populations.

Based on our findings and American College of Sports Medicine guidelines, we recommend that individuals with multiple sclerosis or Parkinson disease perform progressive submaximal strength training (whole-body single and multi-joint resistance exercises) on at least 2 nonconsecutive days per week for an hour under direct supervision (eg, physiotherapist, exercise physiologist, strength and conditioning specialist) to improve muscle strength and other disease specific clinical features (Parkinson disease: mobility and disease progression; multiple sclerosis: fatigue, quality of life, muscle power, maximal electromyography activity, and functional capacity).

### Limitations

Lack of consistent reporting and heterogeneity of study outcomes between trials made it difficult to draw firm conclusions beyond improvements in muscle strength with respect to the benefits of strength training for individuals with multiple sclerosis or Parkinson disease.

## CONCLUSION

Trials investigating the effect of strength training in individuals with Parkinson disease or multiple sclerosis are in their infancy. Nevertheless, benefits in strength were found after strength training in individuals with Parkinson disease and, to a lesser extent, in multiple sclerosis. Some evidence was also found to suggest that strength training has a positive effect on clinical disease progression and mobility in individuals with Parkinson disease. Similarly, some evidence showed that strength training is beneficial for muscle power, maximum electromyography activity, fatigue, functional capacity, and quality of life in individuals with multiple sclerosis. Additional trials employing high-quality methodological designs are required to confirm and expand on these findings. Such trials may provide evidence-based rationale for using strength training as a therapy for other neurodegenerative disorders such as Alzheimer disease and Huntington disease.
